# Disjoint Paired-Dominating sets in Cubic Graphs

**DOI:** 10.1007/s00373-019-02063-w

**Published:** 2019-07-15

**Authors:** Gábor Bacsó, Csilla Bujtás, Casey Tompkins, Zsolt Tuza

**Affiliations:** 1 Computer and Automation Institute, Hungarian Academy of Sciences, Budapest, Hungary; 2 Faculty of Information Technology, University of Pannonia, Veszprém, Hungary; 3 Faculty of Mathematics and Physics, University of Ljubljana, Ljubljana, Slovenia; 4 Alfréd Rényi Institute of Mathematics, Hungarian Academy of Sciences, Budapest, Hungary; 5 Karlsruhe Institute of Technology, Karlsruhe, Germany

**Keywords:** Dominating set, Total dominating set, Paired-dominating set, Claw-free graph, Cubic graph, 05C69

## Abstract

A paired-dominating set of a graph *G* is a dominating set *D* with the additional requirement that the induced subgraph *G*[*D*] contains a perfect matching. We prove that the vertex set of every claw-free cubic graph can be partitioned into two paired-dominating sets.

## Introduction

Except when otherwise specified, we shall deal with simple graphs $G=(V,E)$ with vertex set *V* and edge set *E*. The notions of graph domination, total domination, and paired-domination have been investigated extensively in the literature (see e.g., [4, 5, 7–11] and the references therein). We recall the main definitions.

### Definition 1

A set $D\subseteq V$ is a *dominating set* if every vertex outside *D* has at least one neighbor in *D*. A dominating set *D* is a *total dominating set* if it induces an isolate-free subgraph. A dominating set is *paired-dominating* if it induces a subgraph having a perfect matching.

If *S* is a maximal stable (independent) set in an isolate-free graph *G*, then *S* and $V\setminus S$ are two disjoint dominating sets. On the other hand, Zelinka proved [13] that no lower bound on the minimum degree of *G* guarantees the existence of two disjoint total dominating sets. Since every paired-dominating set is a total dominating set, this theorem implies the same negative result for the (non)existence of two disjoint paired-dominating sets.

Very recently, Desormeaux et al. [6] proved that every claw-free cubic graph has two disjoint total dominating sets, while Akbari et al. [1], independently and simultaneously, proved the same property for a slightly wider subclass of cubic graphs. In this note, we strengthen the result of [6] from another point of view by proving the following theorem.

### Theorem 1

If *G* is a claw-free cubic graph, then its vertex set can be partitioned into two paired-dominating sets.

### Remark 1

This property is stronger than to contain two disjoint paired-dominating sets, in contrast to the case of standard and total domination respectively.

Let us note that Theorem 1 is not a kind of strengthening of the result quoted from [6] that would follow by changing a few words in earlier arguments; in fact it needs a substantially different approach. Namely, the proof in [6] strongly uses hypergraph theorems, while our proof is purely graph-theoretic, with a more specific analysis of the structure. In particular, we first proceed by induction, then define an auxiliary graph and apply Vizing’s theorem to finish the proof.

In Sect. 2, we prove a lemma from which Theorem 1 can be derived easily, as it will be shown in the short Sect. 3.

We now recall a few more notions which will be used in our proof.

### Definition 2

The *open neighborhood* of a vertex *v* is the set of vertices adjacent to *v*. (Thus, it does not contain *v*.)

### Definition 3

For arbitrary graphs *G* and *L*, *G* is *L*-*free* if it does not contain any induced subgraph isomorphic to *L*.

The graph $C_n$ is a chordless cycle on *n* vertices.

The *claw* is a four-vertex three-edge graph with a center and three vertices adjacent to it.

The *paw* is a four-vertex four-edge graph obtained from a claw by adding an edge to it.

The *brill* is a $$C_4$$ having two vertex-disjoint triangles through its two opposite sides ($$V:=\{y,u_1,u_2,z,w_2,w_1\}$$, $$E:=\{w_1u_1,u_1u_2,u_2w_2,w_2w_1,yw_1,$$$$yu_1,u_2z,w_2z\}$$).

The *diamond* is a four-vertex five-edge graph ($V:=\{u,w,y,z\}$, $E:=\{uw,uy,uz,wy,wz\}$). The edge *uw* is called the *central edge* of the (induced) diamond.

A *pair of jewels* is a graph containing two vertex-disjoint induced subgraphs which are one of the diamond and the brill, supplemented by two edges between them so that we obtain a cubic graph.

### Definition 4

A *proper edge-coloring* of a graph is a coloring of its edges such that no adjacent edges receive the same color.

### Remark 2

We shall use other edge-coloring concepts as well.

### Definition 5

The *line graphL*(*H*) of a graph $H=(V,E)$ is the graph with vertex set *E*, where $e,f\in E$ are adjacent in *L*(*H*) if they have a common endpoint in *H*.

### Definition 6

If a vertex has exactly two neighbors and these are non-adjacent, we call it a *cherry vertex*. A triangle is *pendant* if it contains exactly two vertices of degree 2. An edge is *flat* if it is not contained in any triangle. An edge *uv* is a *side-edge* if $d(u)=d(v)=2$ and *u*, *v* are contained in a pendant triangle. In a vertex coloring, an edge is *monochromatic* if its endpoints have the same color.

## Main Lemma

To prove Theorem 1, we intend to use induction on the number of vertices. Along this way, vertices of degree 2 might also occur in the graphs considered. Therefore, we first state a lemma which allows the presence of vertices of degree 2. The vertex partition, that will result in two disjoint paired-dominating sets in case of cubic graphs, is referred to as a 2-coloring of the vertices, say by red (R) and blue (B).

### Lemma 1

If all vertices of the graph *G* have degree 2 or 3 and *G* contains no induced claw and no cherry vertex, then *G* has a vertex 2-coloring such that

1. Both color classes are dominating.
2. The flat edges, side-edges and the central edges of the diamonds are monochromatic in the coloring.

In the following subsections we shall prove Lemma 1. The proof will use induction on *n*, the number of the vertices. We may assume that *G* is connected.

The following statement directly follows from the definitions.

### Claim 1

Assume that all vertices of a graph *G* have degree 2 or 3. Then, *G* is a claw-free graph without cherry vertices if and only if every vertex is contained in at least one triangle. In particular, if such a graph is diamond- and $K_4$-free, a vertex of degree 3 is contained in exactly one triangle and one flat edge.

### Remark 3

The graph $C_n$ shows that the cherry vertex-freeness condition cannot be omitted from the assumptions of the lemma. It is also clear that if *G* contains a vertex *v* of degree 3 which is a center of an induced claw, then no vertex coloring satisfies both Property 1 and Property 2. Hence, the claw-freeness condition cannot be omitted either.

### Reduction of Diamonds and Brills

If the graph *G* contains a diamond or a brill as an induced subgraph, then let *S* be a diamond or a brill in *G*, and omit its vertices in order to apply the induction hypothesis. As *S* is either a diamond or a brill, the following is true: Two non-adjacent vertices, say *y* and *z*, have degree 2 in *S* and all other vertices have degree 3. We shall denote the (only) neighbor of *y* in $V(G)\setminus V(S)$ by $y'$ (if it exists), and $z'$ will be defined similarly. If $V(G)=S$, the validity of the lemma can be checked by hand. If not then at least one of $y'$ and $z'$ exists, say $y'$.

#### Claim 2

$y'\ne z'$, if both exist.

#### Proof

Assume both $y'y \in E(G)$ and $y'z \in E(G)$ hold. This is impossible since if $d_G(y')=2$, then $y'$ would be a cherry vertex in *G*, and if $d_G(y')=3$, we would obtain a claw in *G*, a contradiction.

#### Claim 3

The edge $yy'$ is a flat edge in *G*. Moreover, $y'$ is contained in a triangle in $G[V(G)\setminus V(S)]$. The same is true for $zz'$ (if $z'$ exists).

#### Proof

Observe that *y* and $y'$ do not have a common neighbor, since the two neighbors of *y* in *S* are of degree 3 already inside *S*. Therefore, *y* and $y'$ are not contained in a common triangle and $yy'$ is flat. By Claim 1, $y'$ is incident with a triangle in *G*. Since $yy'$ is flat and $y'$ has no further neighbor in *V*(*S*), this triangle belongs to $G[V(G)\setminus V(S)]$.

We have three cases concerning *y*, *z*, $y'$ and $z'$. In each one, we define a graph $G'$ on the vertex set $V(G')= V(G)\setminus V(S)$, depending on the appropriate case, and prove that $G'$ satisfies the conditions of Lemma 1. With a few exceptions which we handle separately, any vertex coloring *c* of $G'$ can be extended to a vertex coloring of *G* such that Property 1 and Property 2 remain valid (as we shall prove).

**Case 1**$d_G(y)=d_G(z)=3$ and $y'z' \notin E(G)$. To obtain $G'$, we omit the vertices of *S* from *G* and insert the edge $e:=y'z'$. That is, $G'$ will not be an induced subgraph of *G*.

**Case 2**$d_G(y)=d_G(z)=3$ and $y'z' \in E(G)$. In this case, we define $G'$ to be the induced subgraph of *G*, namely $G'= G[V(G)\setminus V(S)]$.

**Case 3**$d_G(y)=3$ and $d_G(z)=2$. Then $G'= G[V(G)\setminus V(S)]$, defined in the same way as in Case 2.

**Proof for Case 1**

In this case, every vertex $v\in V(G')$ has the same degree in $G'$ as in *G*. Hence, $G'$ satisfies the degree condition of Lemma 1. By Claims 1 and 3, every vertex is contained in a triangle in $G[V(G)\setminus V(S)]$ and hence in $G'$ as well. Then, by Claim 1, $G'$ is a claw-free graph without cherry vertices. The connectivity of $G'$ is implied by the presence of the edge *e*.

Consequently, $G'$ satisfies the conditions of Lemma 1 and by the induction hypothesis, there exists a coloring *c* of $G'$ having the properties specified there.

Consider the two triangles $y'w_1w_2$ and $z'w_3w_4$ which are incident with $y'$ and $z'$ in $G[V(G)\setminus V(S)]$. Let us denote $\{w_1,w_2\}$ by *W* and $\{w_3,w_4\}$ by ${\tilde{W}}$. If $W={\tilde{W}}$, then *G* is a pair of jewels, and thus the lemma is clearly true.

#### Remark 4

Here we can observe an exception as mentioned above—not all the colorings can be extended.

Thus, for the equality of the two sets we are done.

Observe that $$|W\cap {\tilde{W}}|=1$$ is impossible since the common vertex would be of degree at least 4, a contradiction. Hence, we may assume that $$w_1,w_2,w_3,$$$$w_4$$ are four different vertices and, as follows, $$y'z'$$ is a flat edge in $$G'$$. Consequently, $$c(y')=c(z')$$ holds.

#### Remark 5

This is the point where it becomes clear why the definition of $G'$ is useful.

We will extend the coloring *c* such that $c(y)=c(y')=c(z')=c(z)$ both for a diamond and a brill. For a diamond, the further vertices of *S* get the opposite color. For a brill, the two flat edges of *S* will be monochromatic, one of them in color red and the other one in color blue.

Property 1 is satisfied since every vertex not in *S* has the same colors in its neighborhood in *G* as it had in its neighborhood in $G'$, and the property can be checked directly for the vertices in *S*.

Property 2 also holds for this extended coloring because each flat, central or side-edge of *G* either has a vertex from *V*(*S*), and the extension rule of *c* ensures its monochromaticity; or it was a flat, central or side-edge already in $G'$ and remained monochromatic in the coloring of *G*.

**Proof for Case 2**

Similarly as in Case 1, our goal is to reach a situation where in the coloring *c* of $G'$ which we wish to extend, we have $c(y')=c(z')$.

By Claim 3, $y'$ is contained in a triangle of $G'$ and so is $z'$. By the degree conditions for *G*, these two tringles coincide. Let us call the third vertex *w*.

The connectivity of $G'$ holds, and $G'$ satisfies all the other conditions of Lemma 1. If $G'$ consists of only the triangle $T:=y'z'w$, then we can clearly find the required coloring of the whole graph, both for the diamond and the brill.

#### Remark 6

We can observe again the exception mentioned above.

Otherwise *T* is a pendant triangle with the side-edge $y'z'$ in $G'$. Then, by the hypothesis, there exists a coloring *c* satisfying Property 1 and Property 2 for $G'$ and, in particular, $c(y')=c(z')$. We are in the desired situation, and we are done for this case.

#### Remark 7

Note that $y'z'$ is no longer a side-edge in *G*.

**Proof for Case 3**

By Claim 3, the vertex $y'$ is contained in a triangle in $G'$ and has $d_{G'}(y')=2$. With reasoning similar to that in Case 2, one can prove that $G'$ satisfies the conditions of the lemma. By the induction hypothesis, there exists a coloring *c* in $G'$ that satisfies the desired properties. If $G'$ consists of only one triangle $y'ww'$, then $ww'$ is a side-edge in *G*; therefore we specify *c* such that $c(w)=c(w')\ne c(y')$. Otherwise, we may take any appropriate coloring *c*. To extend *c*, we fix $c(y)=c(z)=c(y')$, while the further vertices of the diamond or brill are colored as described in Case 1.

The applicability of the induction hypothesis and the desired properties of the coloring constructed have been proved for the case when a diamond or brill exists in *G*.

### Reduction of Degree-2 Vertices

From this point on, we suppose that *G*, which satisfies the conditions of Lemma 1, is diamond- and brill-free. Throughout this subsection, we also use the induction hypothesis that Lemma 1 holds for every graph $G'$ with $|V(G')| < |V(G)|$. Suppose there exist vertices of degree 2 in *G*. The smallest among such graphs is $K_3$; Lemma 1 is valid for it. We may suppose we have some larger graph in hand, we pick a vertex *x* of degree 2. Let *y* and *z* be its neighbors. Since *G* contains no cherry vertex, $yz\in E$. If *y* has degree 3, we denote its third neighbor by $y'$, and $z'$ will be defined similarly. Since $d_G(x)=2$ and *G* is diamond-free, $y'$ is adjacent to neither of *x* and *z*. The analogous statement is true for $z'$. We obtain that $y'\ne z'$ and moreover, $yy'$ and $zz'$ are flat edges (if they exist). Together with Claim 1, this implies that $y'$ ($z'$) is incident with a triangle in $G'=G[V(G)\setminus \{x,y,z\}]$.

**Case 1** Suppose that $d_G(y)=d_G(z)= 3$. Take the graph $G'$, obtained by deleting the vertices *x*, *y*, *z* from *G*. Since every vertex from $V(G')$ is incident with a triangle in $G'$, we may conclude by Claim 1 that $G'$ satisfies the conditions of Lemma 1. Consequently, the induction hypothesis can be applied for $G'$. We take a coloring *c* of the vertices in $G'$ satisfying Properties 1 and 2. If there is a triangle which is a component in $G'$ and becomes a pendant triangle in *G*, then *c* is specified such that the side-edge of *G* is monochromatic already in the coloring of $G'$. We shall extend *c* to a valid coloring of *G*.

#### Remark 8

Here is an exception again, as mentioned above.

We define $c(y):=c(y')$ and $c(z):=c(z')$. If $c(y)=c(z)$, then we color *x* by the opposite color, otherwise we color *x* arbitrarily. We claim that the coloring constructed for *G* satisfies all of the required conditions. Both color classes are dominating since we have that the three omitted vertices are dominated by the opposite color and the vertices in $G'$ inherit this property. We observe that the flat edges $yy'$ and $zz'$ are monochromatic in *c*, and all the further flat edges of *G* were flat edges already in $G'$. Hence, Property 2 also holds for the extended coloring *c*.

**Case 2** Suppose that $d_G(y)= 3$ and $d_G(z)= 2$. The argument in this case is similar, so we omit it. The only difference is that here *xz* is a side-edge. Hence, after we set $c(y):=c(y')$, the colors of *z* and *x* are determined as $c(x)=c(z)\ne c(y)$.

### The Last Step: A Direct Proof

We may suppose already that *G* is cubic and, moreover, $K_4$-, claw-, diamond- and brill-free. We are in the position to complete the proof of Lemma 1. (Further usage of the inductive hypothesis is not required.)

#### Proposition 1

If *G* is a claw- and diamond-free graph, then it is isomorphic to the line graph *L*(*H*) of some simple graph *H*.

#### Proof

This follows directly from [2, 3] as all of the forbidden induced subgraphs described there, other than the claw, contain a diamond.

#### Remark 9

We may suppose that *H* is connected.

#### Claim 4

*H* is triangle-free.

#### Proof

Let us assume (by way of contradiction) that there is a triangle *U* in *H*. By the previous remark, there exists an edge between *V*(*U*) and $V(H)\setminus V(U)$. If there are no additional edges, then we obtain a paw graph. Its line graph is a diamond, a contradiction. Otherwise, with more edges, we obtain a graph having a paw as a (non-induced) subgraph and thus *G* contains a diamond again. This completes the proof of the claim.

#### Definition 7

Let $S_3$ be the set of degree-3 vertices in *H* and let $T:=V(H)\setminus S_3$.

#### Claim 5

The following properties hold.

- *H* has no vertex of degree at least 4.
- $S_3$ is a stable set.

#### Proof

Since $G=L(H)$ is a $K_4$-free cubic graph, both properties are valid.

#### Claim 6

*T* satisfies the following properties.

- Every vertex of *H* in the set *T* has degree 2.
- *T* is a stable set.

#### Proof

First, suppose that some vertex $t\in T$ has degree 1 in *H*. By Claim 5, the maximum degree of *H* is at most 3 and therefore, the only edge containing *t* would yield a vertex of degree of at most 2 in *G*. This contradiction establishes *a*). Next, assume that an edge $e=xy$ is contained in *T*. Then, since $d_H(x)=d_H(y)=2$, the vertex of *G* representing *e* would be of degree 2 which contradicts our assumption on the 3-regularity of *G*.

#### Proposition 2

*H* is bipartite.

#### Proof

This follows from Claims 5b and 6b.

Using the auxiliary statements above, finally we distinguish three cases.

**Case 1** If in the graph *H*, some pair of vertices in $S_3$ have three common neighbors then, by connectivity, *G* is just a graph with two vertex-disjoint triangles and a 1-factor, and the lemma is valid for this graph.

**Case 2** Some pair of vertices in $S_3$ with two common neighbors would yield a brill in *G*, a contradiction.

**Case 3** Suppose every pair of vertices in $S_3$ has at most one common neighbor in *T*. We need to introduce a new definition. (The proof of this case is continued below the proof of Proposition 3.)

#### Definition 8

An *edge-quasi-coloring* of a graph *A*, having minimum degree at least 2, is a coloring of its edges by two colors with the condition that in every vertex of *A*, both edge colors can be found.

#### Proposition 3

Every cubic graph has an edge-quasi-coloring.

#### Proof

By Vizing’s Theorem [12], any cubic graph has a proper 4-edge-coloring with colors *R*1, *R*2, *B*1, *B*2. We unify the colors *R*1, *R*2 to one color, red, and similarly, we take another color, blue, for the color classes *B*1, *B*2. It is obvious that we obtain an edge-quasi-coloring.

In Case 3, by Claim 6, *H* can be considered as a graph obtained from a cubic auxiliary graph *A* in the following way: every edge of *A* is subdivided with one additional vertex.

Now we take an edge-quasi-coloring of *A* by applying Proposition 3. When we subdivide an edge *e*, the colors of the two new edges are defined to inherit the color of *e*. Clearly, this coloring yields a vertex coloring in *G* such that both color classes are dominating sets in *G*. Thus the coloring satisfies Property 1. A pair of adjacent edges in *H* yields a flat edge *e* in *G* if and only if their common endpoint is in *T*. The edge coloring of *H*, constructed above, ensures that *e* is monochromatic in the coloring of *G* and therefore, every flat edge is monochromatic. By our present assumption, *G* is cubic and diamond-free and consequently, it contains neither central nor side-edges. These prove that the coloring satisfies Property 2 as well.

We have completed the proof of Lemma 1.

## Proof of Theorem 1

It suffices to prove Theorem 1 only for connected graphs. Also, the assertion clearly holds for $G\cong K_4$.

Consider a claw-free, cubic and connected graph $G\not \cong K_4$. As it satisfies the conditions of Lemma 1, *G* admits a coloring *c* with Properties 1 and 2. We may have two types of vertices $v\in V(G)$. If the open neighborhood of *v* contains exactly one edge, *v* is incident with exactly one triangle and one flat edge. If the open neighborhood contains exactly two edges (induces a $P_3$), *v* is contained in two triangles and is incident with the central edge of an induced diamond. In the latter case, *v* cannot be contained in any flat edges. These imply that the set of flat- and central edges form a perfect matching in *G*. Property 2 ensures that all of these edges are monochromatic and therefore, each of the two color classes contains a perfect matching. By Property 1, the color classes are dominating sets as well. Consequently, the 2-coloring *c* partitions *V*(*G*) into two paired-dominating sets. This establishes Theorem 1.

## Concluding Remarks and Open Problems

The proof of the main lemma—and hence also that of Theorem 1—is constructive and can be easily modified to give a polynomial-time algorithm for finding the color classes and (paired-) dominating sets desired.

We present here a natural potential extension of the main lemma. Namely, we would like to omit the cherry vertex-freeness condition in this modified situation. For a cherry vertex *y* with neighbors *x* and *z*, the edges *xy* and *yz* would be monochromatic because of Property 2. Thus, even the domination property would disappear for one of the color classes. From this phenomenon, the following open problem arises:

### Problem 1

Is the following statement true?

For a claw-free graph, having only vertices of degree 2 or 3, there exists a vertex coloring satisfying the following two properties:

1. Both color classes are dominating.
2. A vertex of degree 3 is never isolated in its own color class.

There are further questions which arise concerning Theorem 1 in a natural way.

### Problem 2

Give necessary or sufficient conditions on the structure of graphs *G* for each of the following properties to hold.

1. *G* contains two disjoint paired-dominating sets;
2. *G* admits a vertex partition into two paired-dominating sets.

### Problem 3

Given a graph *G* without isolated vertices,

1. estimate the maximum number of mutually disjoint paired-dominating sets contained in *G*;
2. estimate the maximum number of paired-dominating sets into which *V*(*G*) can be partitioned, if such a partition exists.

The graph invariant asked for in Problem 3(1) is always positive because every non-extendable matching induces a paired-dominating set whenever isolates are excluded.
